# 5-Fluorouracil-Loaded Folic-Acid-Fabricated Chitosan Nanoparticles for Site-Targeted Drug Delivery Cargo

**DOI:** 10.3390/polym14102010

**Published:** 2022-05-13

**Authors:** Shafi Ullah, Abul Kalam Azad, Asif Nawaz, Kifayat Ullah Shah, Muhammad Iqbal, Ghadeer M. Albadrani, Fakhria A. Al-Joufi, Amany A. Sayed, Mohamed M. Abdel-Daim

**Affiliations:** 1 Advanced Drug Delivery Lab, Gomal Center of Pharmaceutical Sciences, Faculty of Pharmacy, Gomal University, Dera Ismail Khan 29050, Pakistan; shafikustian@gmail.com (S.U.); asifnawaz676@gmail.com (A.N.); kifayatrph@gmail.com (K.U.S.); iqbalmiani@gmail.com (M.I.); 2 Pharmaceutical Technology Unit, Faculty of Pharmacy, AIMST University, Bedong 08100, Malaysia; 3 Department of Biology, College of Science, Princess Nourah bint Abdulrahman University, Riyadh 11671, Saudi Arabia; gmalbadrani@pnu.edu.sa; 4 Department of Pharmacology, College of Pharmacy, Jouf University, Sakaka 72341, Saudi Arabia; faaljoufi@ju.edu.sa; 5 Zoology Department, Faculty of Science, Cairo University, Giza 12613, Egypt; amanyasayed@sci.cu.edu.eg; 6 Department of Pharmaceutical Sciences, Pharmacy Program, Batterjee Medical College, Jeddah 21442, Saudi Arabia; 7 Pharmacology Department, Faculty of Veterinary Medicine, Suez Canal University, Ismailia 41522, Egypt

**Keywords:** colon cancer, targeted delivery, folate-conjugated nanoparticles, cytotoxicity

## Abstract

Nanoparticles play a vital role in cancer treatment to deliver or direct the drug to the malignant cell, avoiding the attacking of normal cells. The aim of the study is to formulate folic-acid-modified chitosan nanoparticles for colon cancer. Chitosan was successfully conjugated with folic acid to produce a folic acid–chitosan conjugate. The folate-modified chitosan was loaded with 5-FU using the ionic gelation method. The prepared nanoparticles were characterized for size, zeta potential, surface morphology, drug contents, entrapment efficiency, loading efficiency, and in vitro release study. The cytotoxicity study of the formulated nanoparticles was also investigated. The conjugation of folic acid with chitosan was confirmed by FTIR and NMR spectroscopy. The obtained nanoparticles were monodispersed nanoparticles with a suitable average size and a positive surface charge. The size and zeta potential and PDI of the CS-5FU-NPs were 208 ± 15, 26 ± 2, and +20 ± 2, respectively, and those of the FA-CS-5FU-NPs were 235 ± 12 and +20 ± 2, respectively, which are in the acceptable ranges. The drug contents’ % yield and the %EE of folate-decorated NPs were 53 ± 1.8% and 59 ± 2%, respectively. The in vitro release of the FA-CS-5FU-NPs and CS-5FU-NPs was in the range of 10.08 ± 0.45 to 96.57 ± 0.09% and 6 ± 0.31 to 91.44 ± 0.21, respectively. The cytotoxicity of the nanoparticles was enhanced in the presence of folic acid. The presence of folic acid in nanoparticles shows much higher cytotoxicity as compared to simple chitosan nanoparticles. The folate-modified nanoparticles provide a potential way to enhance the targeting of tumor cells.

## 1. Introduction

Colon cancer (CC) is one of the leading causes of mortality and morbidity in the world. Nine percent of all cancer cases are colon cancer. Throughout the world, CC is the third most common cancer type and the fourth most common cause of death [1]. CC is the most common type of cancer among all cancer types in western countries. Cases of colorectal cancer have increased remarkably in the past fifty years, and such cases have become the second highest in females and the third highest in males. The primary means of treatment for CC is surgery, with chemotherapy and/or radiotherapy being indicated depending on the nature and severity of the disease. Chemotherapy using different anticancer agents can be used as an adjuvant treatment on the second number after surgery, as a neo-adjuvant treatment before surgery, or as a main treatment to abate tumor size and growth as well as metastasis risk [2]. The 5-fluorouracil (5-FU) drug is an analog of the pyrimidines, and therefore uses the same metabolic routes as uracil and thymine. It is classified as an antimetabolite drug, interfering with nucleoside metabolism in RNA and DNA, and is used for the treatment of various tumors, such as those found in breast adenocarcinoma, the gastrointestinal tract, the ovary, the head, and the neck [3]. Despite its effectiveness, 5-FU presents some drawbacks. After oral administration, 5-FU bioavailability is highly variable due to its inconsistent absorption in the gastrointestinal tract and first-pass metabolism through the liver. Thus, the 5-FU half-life is extremely short (6–20 min), and frequent and high doses are required to maintain adequate plasma concentrations [4]. An alternative to overcoming these drawbacks and improving drug bioavailability, promoting controlled drug release, and choosing for more cell selectivity is the application of polymeric nanoparticles as 5-FU carriers. The oral delivery of drugs is of tremendous interest for patients seeking safe and controlled drug delivery. Compared to injections, the oral administration of anticancer drugs via oral route is cost-effective, reducing the hospitalization duration of the patient, as well as improving the patient’s quality of life. Examples of some drugs that are used for cancer treatment are as follows: 5-flourouracil (5-FU), hexacarbonyl-(5-FU), and N^4^ pentoxylcarbonyl-5-deoxy-5flourocytidine (capecitabine) [5].

Chitosan is a semi-crystalline, linear polysaccharide that is composed of (1-4)-2-acetamido-2-deoxy-β-D-glucan (*N*-acetyl D-glucosamine) and (1-4)-2-amino-2-deoxy-β-D-glucan (D-glucosamine) units. Chitosan is not extensively present in the environment in its original form but can be derived easily from chitin (a natural polymer) by removing its acetyl group. The ratio of D-glucosamine to the sum of D-glucosamine and N-acetyl D-glucosamine gives the degree of deacetylation (DD) of chitosan. DD indicates the number of amino (NH_2_) groups along the chains [6]. Chitosan provides a valuable tool for the current system of novel drug delivery owing to its intrinsic biological and physicochemical properties. The characteristics of chitosan nanoparticles (NPs), such as their small size, better stability, inexpensiveness, easy manufacturing process, lower toxicity, and versatile method of administration, made them favorable drug and gene delivery carriers. Chitosan can be easily chemically modified due to the presence of its active functional groups such as amine (NH_2_) and hydroxyl (OH) groups. Due to pH changes and electrostatic interactions throughout the gastrointestinal tract (GIT) that are vital for maintaining the NP’s stability, the permanent positive charge of chitosan favors their mucoadhesion property in the intestinal mucosa layer. This characteristic has been used to develop enhanced drug delivery systems that could help in CC treatment [7].

The surface morphology of nanoparticles (NPs) can be modified with the conjugation of targeting ligands, such as folic acid (FA), antibodies, integrins, transferrin, and polysaccharides, to improve receptor affinity and internalization by target tissues. Many tumor cell surfaces overexpress folate receptors (FRs), which are less often expressed in normal and healthy cells. This feature makes tumor cells an excellent target for tumor-targeting drug delivery [8]. Folic acid (FA) has emerged as an optimal targeting ligand for the selective delivery of attached imaging and therapeutic agents to cancer cells and inflammation sites. The use of FA as a target ligand has arisen primarily from its following features: (1) its easy conjugation to both therapeutic and diagnostic agents; (2) its great affinity for the folate receptors (FRs); and (3) the distribution of folate receptors (FRs) in limited numbers in normal tissues. Folic acid as a targeting ligand has been investigated by many scientists [9]. In one study, it was demonstrated that the folic-acid-modified chitosan NPs were excellent vectors for the colon-specific delivery of 5-aminolevulinic acid (5-ALA) for fluorescent endoscopic detection [10]. The FA decoration upheld the establishment of a genuine affinity for FRs+ cancer cells even when co-cultured closely with higher numbers of healthy cells [11]. In one study, FA was evaluated in vitro, in which it was conjugated with carboxymethyl chitosan, and its nanoparticles were loaded with doxorubicin for targeted drug delivery. These FA-modified NPs manifested FA feasibility as an excellent targeted delivery carrier [12]. In the present study, folic-acid–chitosan-conjugated nanoparticles for oral delivery were prepared and evaluated for in vitro release and cytotoxicity studies.

## 2. Materials and Methods

### 2.1. Materials

Chitosan (deacetylation degree—83% and mol wt—310,000–375,000), 5-flourouracil and folic acid were obtained from Sigma-Aldrich (lot# A263299) (Sigma-Aldrich, Inc. St. Louis, MO, USA). TPP (85%), potassium dihydrogen phosphate, calcium chloride, 1-ethyl-3-(3 dimethylaminopropyl) carbodiimide (EDC), and sodium hydroxide were obtained from Sigma Chemicals (Merck Pte. Ltd. 2 Science Park Drive, Singapore). Acetic acid, hydrochloric acid, ethanol, and DMSO were obtained from Merck (Merck KGaA, Darmstadt, Germany).

### 2.2. Conjugation of Folic Acid (FA) with Chitosan (CS)

The conjugation process of folic acid (FA) to chitosan (CS) is described as follows. FA and 1-ethyl-3-(3-dimethylaminopropyl) carbodiimide (EDC) solution in anhydrous dimethylsulfoxide (DMSO) (20 mL), with 1:1 molar ratio, was made and stirred at room temperature until the EDC and FA were mixed well. The solution was then slowly added to 0.5% (*w*/*v*) CS in an aqueous solution of 0.1 M of acetic acid with a pH of 4.7, and then stirred at 25 °C in the dark area for 16 h to let the FA conjugate onto the CS molecules. Then, 1 M of NaOH was added to adjust the pH of the solution to 9.0. The solution was centrifuged at 2500 rpm to settle down the FA–CS conjugate. The sediment was first dialyzed against a phosphate buffer with a pH of 7.4 for 3 days, and then against water for 4 days. Finally, the FA–CS conjugate was collected as a sponge by freeze-drying and kept for further study [13].

#### 2.2.1. Fourier Transform Infrared Spectroscopy

The Fourier transform infrared spectroscopy (FTIR) was performed using an ATR FTIR spectrometer (L1600300, PerkinElmer, Beaconsfield, UK). The FTIR spectra of chitosan, folic acid, and its conjugate (FA–CS) were obtained. The recording range of the spectrum was 600–4000 cm^−1^ at 32 scans per minute with a resolution of 4 cm^−1^ in absorbance mode. After recording, the spectra were baseline, corrected, and normalized using Spectra software to identify the characteristic peaks and differences [14].

#### 2.2.2. H-NMR

For NMR spectroscopic analyses, solutions of CS, FA, and their conjugates were prepared in 1.97 mL of CDCl_3_ and kept at room temperature until their complete dissolution. Acetic acid was used as a co-solvent for the solubility of chitosan in CDCl_3_. ^1^H-NMR spectra were obtained using a Bruker AV-500 MHz NMR spectrometer. Bruker–Topspin software (version 4.1.1) was used for the analysis of NMR spectra.

#### 2.2.3. Determination of Folate (FA) Content

The FA–CS conjugates were accurately weighed and then dissolved in 50 mL of 0.2 molar sodium bicarbonate buffer solution with a pH of 10 at 25 °C with magnetic stirring. The solution was centrifuged at 3500 rpm for 10 min (Laboratory Centrifuge, YJ03-0434000, Shanghai, China). The supernatant was tested for determining folate (FA) using a UV–visible spectrophotometry technique with a wavelength of 365 nm. The folate content was calculated as the percentage of FA in a unit weight of conjugate. For each experiment, at least three duplicates were carried out and the results were averaged.

### 2.3. Preparation of Nanoparticles (NPs)

The FA–CS NPs were synthesized by ionic cross-linking with tripolyphosphate (TPP) using the method described by Salar and Kumar, 2016, with slight modifications. The FA–CS conjugate solution (0.2%, *w*/*v*, pH 2.5) was prepared using 1% *v*/*v* acetic acid at room temperature. The TPP (0.2%, *w*/*v*) solution in distilled water was prepared. For the synthesis of the 5-FU-loaded nanoparticles, an aqueous solution of 5-FU (500 mg/10 mL) was prepared separately. A solution of 5-FU was added drop-wise into the FA–chitosan conjugate solution. The TPP solution was added into the conjugate solution drop-wise in a 1:3 ratio. The solution was allowed to stir for 1 h on a magnetic stirrer at room temperature. The nanoparticle’s suspension was centrifuged at 5000 rpm for 10 min for separating the nanoparticles from the solution, and then freeze-dried for 24 h to obtain the final product of the NPs. NPs without an FA conjugation were also prepared in the same manner.

### 2.4. Characterization

#### 2.4.1. NP Size and Zeta Potential

Photon correlation spectroscopy was used to determine the particle size and zeta potential of FA-CS-5FU-NPs and CS-5FU-NPs at 25 °C in quartz cell and zeta potential cell with a detect angle of 90°, respectively, using a Malvern Zetasizer Nano ZS 90 (Malvern Instruments Ltd., Malvern, UK). In 5.0 mL of deionized water, one mg of NPs was added, and vortex stirring (Velp Scientifica, Usmate Velate, Italy) was used to fortify the mixture [15].

#### 2.4.2. Nanoparticle Morphology

The surface morphology of NPs was examined using the scanning electron microscopy (SEM) technique (JSM6360LA, JEOL, Tokyo, Japan). The NPs were fixed with carbon tape onto studs and directly examined under the SEM. Images of the NPs were captured at a 20,000× magnification level [15].

#### 2.4.3. Percentage Yield, Drug Entrapment Efficiency (%EE), and Drug-Loading Efficiency

Precisely weighed 15 mg of FA-CS-5FU-NPs and CS-5FU-NPs was dispersed in 15 mL of distilled water under magnetic stirring at 200 rpm for 2 h in two separate beakers followed by centrifugation (Laboratory Centrifuge, YJ03-0434000, Shanghai, China) at 5000 rpm for 45 min. The supernatant of both formulations was isolated and analyzed for free 5-FU using the UV spectroscopy technique. The percentage (%) yield was calculated using the formula given in Equation (1) [16]:

*% yield* = Mass of NPs obtained/ total weight of drug and polymer × 100 (1)

Both drug entrapment efficiency (%EE) and drug-loading efficiency were determined indirectly using free drug concentration. After centrifugation, the obtained sediments of the formulations were dissolved in ethanol, aliquot filtered, and analyzed at 265 nm in UV spectroscopy for drug entrapment efficiency using Equation (2).

(2) $$EE\;\left(\%\right)=\frac{5\mathrm{FUtotal}-5\mathrm{FU}\;\mathrm{Free}}{5\mathrm{FUtotal}}\times 100$$

The total drug load collected from the supernatant and sediment was used to calculate the drug-loading efficiency using Equation (3). Triplicates were conducted and the results were averaged [16].

(3) $$Drug\;loading\;efficiency=\frac{5\mathrm{FUtotal}-5\mathrm{FUfree}}{\mathrm{Weight}\;\mathrm{of}\;5\mathrm{FU}\;\mathrm{loaded}\;\mathrm{nanoparticles}\;\mathrm{taken}}\times 100$$

### 2.5. In Vitro Release of Nanoparticles

The release rate for the designed formulations was studied for up to 2 h in 900 mL of release media such as simulated gastric fluid (solution of 0.2 MHCL and 0.2 MKCl, pH 1.2) and simulated intestinal fluid (solution of 0.2 M potassium dihydrogen phosphate and 0.1 M sodium hydroxide, pH 6.5) for up to 24 h using a dissolution tester (basket method type 1) at 37.5 ± 0.5 °C. The stirring speed was set at 100 rpm. Then, 15 mg of FA-CS-5FU-NPs and CS-5FU-NPs was placed in two separate baskets and run the apparatus. At predetermined time intervals (0.5, 1, 1.5, 2, 4, 8, 12, 16, 20, and 24 h), a 5 mL sample was withdrawn and replaced with a fresh dissolution medium. All the samples were analyzed using a UV–visible spectrophotometer at a wavelength of 265 nm. The cumulative percentage of the drug released was calculated [17].

### 2.6. Cytotoxicity Studies

For the cytotoxicity study, human colon carcinoma cell lines (Caco2) were used. Cells were cultured in Eagle’s minimum essential medium supplemented with 2 mM of glutamine, 20% fetal bovine serum (FBS), 1.5 g/L of sodium bicarbonate, 1 mM of sodium pyruvate, and 0.1 mM of nonessential amino acid. Cells were equilibrated with 5% CO_2_. Growing temperature was set at 37 °C in an incubator allowing humidified air to pass through. Cytotoxicity studies of the 5-FU solution, CS-5-FU-NPs, and FA-CS-5-FU-NPs were performed on the Caco-2 using the MTT (3-(4,5-dimethylthiazol-2-yl)-2,5-diphenyltetrazolium bromide) assay. First, 5 × 10^3^ cells were seeded in 96-well plates and incubated for 24 h without drug/formulations. The cells were then treated with the 5-FU solution, CS-5FU-NPS, and FA-CS-5FU-NPS, and were then incubated for 24 h. The cells in the absence the 5-FU solution, CS-5FU-NPS, and FA-CS-5FU-NPSNPs were considered as the control group. The MTT solution was added to assess the cytotoxicity of drug and nanoparticles. Cells were incubated for 4 h in MTT solution followed by the addition of DMSO to dissolve formazan and quantified spectrophotometrically using a microplate reader (Thermo Varioscan Multiplate Reader).

### 2.7. Data Analysis and Statistics

The obtained data were statistically analyzed using ANOVA (one-way analysis of variation) and the student’s *t*-test (IBM^®^ SPSS^®^ Statistics version 19, Armonk, NY, USA), and the Statistical Package Minitab^®^ version 20 (Minitab, LLC, State College, PA, USA). Data with values of *p* < 0.05 were considered statistically significant. All the tested data were described as triplicate (*n* = 3) and mean ± standard deviation (S.D.).

## 3. Results and Discussion

### 3.1. Synthesis of FA–CS Conjugate

**Figure 1:**
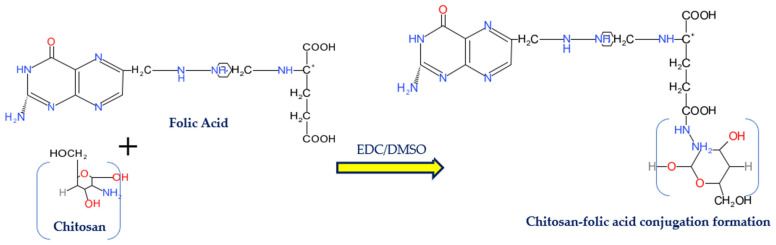
A scheme illustrating the reaction of chitosan with folic acid.

**Figure 2:**
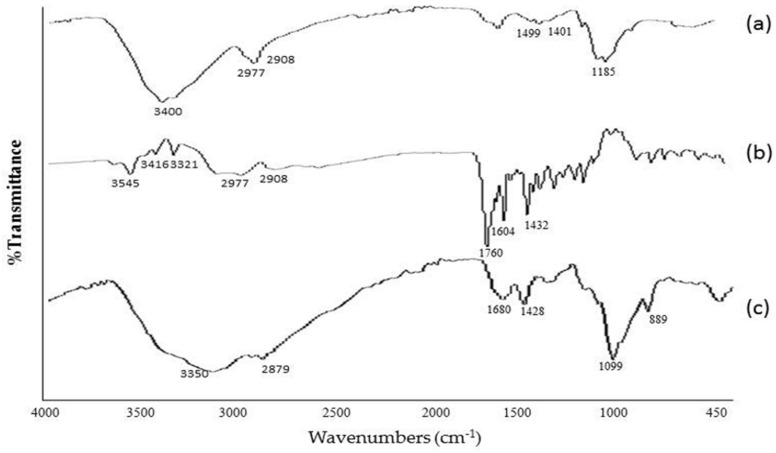
FTIR spectra of (**a**) pure chitosan, (**b**) pure FA, and (**c**) FA–CS conjugate.

**Figure 3:**
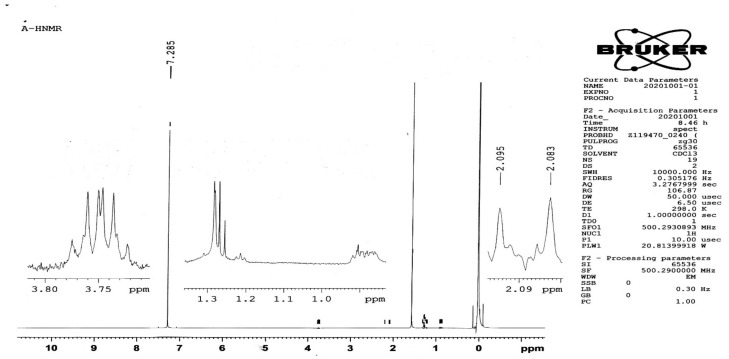

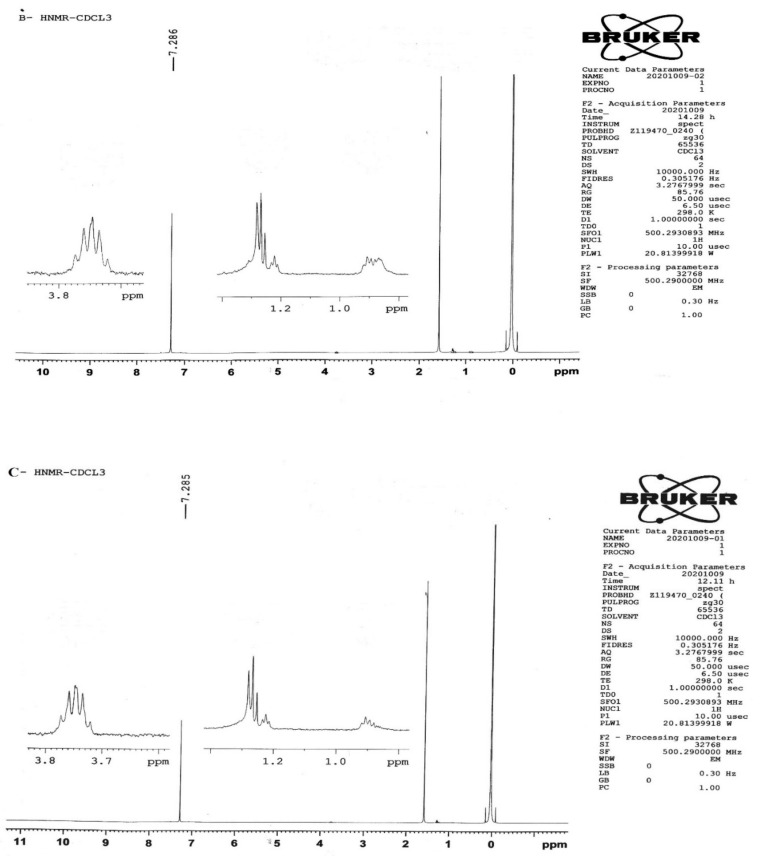
^1^H-NMR spectra of (**A**) pure chitosan, (**B**) pure FA, and (**C**) FA–CS conjugate.

The synthesis of the FA–CS conjugates was carried out by means of carbodiimide chemistry using the water-soluble 1-ethyl-3-(3-dimethylaminopropyl) carbodiimide (EDC) (Figure 1). The EDC is a “zero-length” crosslinked chemical. It is used in the formation of conjugate via amide linkage without leaving a spacer molecule [18]. The EDC reacted with the COO^−^ of the FA and 5-FU to form an intermediate of active ester. The intermediate reacted further with the primary amine (NH_2_) group of the CS, giving rise to an amide (N–H) bond, with an isourea by-product that was removed easily by filtration or dialysis [18]. The FTIR and ^1^H-NMR spectra (Figure 2 and Figure 3) successfully confirmed the conjugation of folic acid onto chitosan molecules.

#### 3.1.1. FTIR Studies

In an FTIR of chitosan (Figure 2), a strong band at the region of 3400 cm^−1^ represents NH functional groups (primary amine). The absorption band at around 2977 cm^−1^ can be attributed to CH symmetric stretching. Bands at 1401 cm^−1^ indicate a methyl group (CH_3_). Symmetrical bending in the range of 1260–800 cm^−1^ belong to the glycosidic ring; in particular, the band at 1156 cm^−1^ corresponds to the glycosidic linkage. Similarly, an FTIR of pure folic acid showed the IR spectrum at 3100–3500 cm^−1^ which can be attributed to the OH carboxylic of glutamic acid moiety and the NH group of the pterin ring stretching. Absorption at 1760 cm^−1^ represents C=O carboxylic acid in pure FA. Similarly, the absorption band at 1432 cm^−1^ represents the phenyl and the pterin ring. The band at 3321 cm^−1^ pure folic acid is absent/overlapped in the conjugate formulation, indicating the coupling of folate with a chitosan polymer [19]. The primary amine of the chitosan reacted with the carboxylic acid group of folic acid, forming an amide bond. The amide bond formation between the chitosan and folic acid was evidenced by a shift of the FTIR wavenumber of folic acid from 1760 to 1680 cm^−1^. The assignment of FTIR peaks was correlated with earlier studies [20,21,22]. The peaks at 2.07 ppm attributed to the acetamino group CH3, and the CH peak appeared at 3.50–3.95 ppm, corresponding to carbons 3, 4, 5, and 6 of the glucosamine rings of CS.

#### 3.1.2. H-NMR Study

FA has two active –COOH groups at its end point. Among these, γ-COOH is more sensitive to the reaction, owing to its high reactivity [23]. The final product of FA-CS was synthesized by the reaction between the activated FA ester and the primary amine NH_2_ groups of CS through the formation of an amide bond under homogeneous conditions. The peaks at 2.08 ppm attributed to the hydrogen atom of the methyl group (CH_3_) of the acetamino groups of chitosan, as well as CH peaks at 3.77–3.8 ppm, can be explained by hydrogen bonded to carbons 3, 4, 5, and 6 of the glucosamine rings of CS [24]. The CS conjugation was confirmed by the peculiar signals at 2.5 ppm, which attributed to the aromatic protons of the FA, and characteristic peaks at 2.84 ppm corresponded to the FA proton from the H22 [25]. This was ascribed to the development of amide linkage after the folic acid–chitosan conjugation. Ji et al. previously reported similar results at 2.45 ppm in relation to the FA proton from the H10 and H22 [24], respectively, which is in line with the current study.

#### 3.1.3. Folate (FA) Content

Folate content was found to be 5% of the total weight of the FA-CS-5FU-NPs formulation. Folic acid is commonly engaged as a ligand for targeting cancer cells, as its receptors are over-expressed on the surface of several human cancer cells. Integrating folic acid into chitosan-based drug delivery inventions directs the systems with a well-organized targeting ability [26].

### 3.2. Characterization of Nanoparticles

#### 3.2.1. Size, Zeta Potential, and Surface Morphology

**Figure 4:**
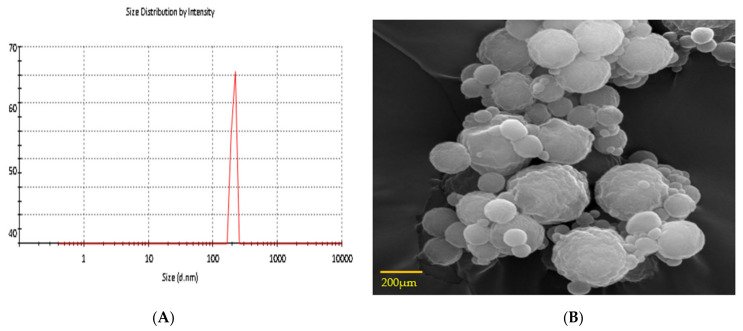
(**A**) Size distribution of nanoparticles, (**B**) surface morphology of folic-acid-modified 5-FU-loaded chitosan NPs.

The size of the CS-5-FU NPs was found to be 208 ± 14.65 nm, while the FA-CS-5-FU NPs was 235 ± 11.5 nm, as shown in Table 1 and Figure 4A,B. The poly dispersity index (PDI) was found to be 0.2 and 0.19 for of the FA-CS-5-FU NPs and the CS-5-FU NPs. This small size of NPs is important since such NPs in anticancer drugs can easily escape the leaky tumor vasculature and accumulate within the tumor region to exert cytotoxic effects on proliferating cells [27]. The zeta potential of the FA-CS-5-FU NPs was found to be +20 ± 2. The FA-CS-5-FU NPs show an insignificant decrease in zeta potential as compared to the CS-5-FU NPs (Table 1; *p* > 0.05). Other studies have also found a decrease in the value of zeta potential after folate conjugation. [28,29,30]. The obtained zeta potential value of +26 ± 2 mV in the instance of folic-acid-modified chitosan NPs indicates that folic acid binds to chitosan quite strongly. The free positive NH_2_ groups of chitosan molecules may account for the positive value. This positive zeta potential is helpful in crossing the negatively charged membrane of cancer cells. The value of the zeta potential (ZP) indicates the repulsive interactions between suspended particles and can therefore be used to forecast the stability of colloidal aqueous dispersions. The prepared nanoparticles were spherical in shape and smooth in surface, as shown in Figure 4B.

#### 3.2.2. Drug Content, Encapsulation Efficiency, and Drug-Loading Efficiency

TPP was used as a cross linker in folate-modified chitosan nanoparticles loaded with 5-FU. The drug content and %EE of the drug were estimated based on the amount of the drug in the supernatant and the sedimented pellets of dispersed nanoparticles after centrifugation. The FA-CS-5-FU NPs demonstrated 5-FU content of 53 ± 0.14% and EE of 59 ± 0.23%. The drug-loading efficiency was 43 ± 3% and 39 ± 2% for CS-5-FU NPs and FA-CS-5-FU NPs, respectively. A decrease in the loading efficiency of NPs with FA conjugation occurred because the folic acid changed a number of amino groups on the chitosan molecules, lowering their positive charges and thereby attracting drug molecules [13]. Consequently, it emerged that the amount of folic acid conjugations in the mixture had a significant effect on the loading efficiency (LE) (Table 1; *p* < 0.05).

### 3.3. In Vitro Release

**Figure 5:**
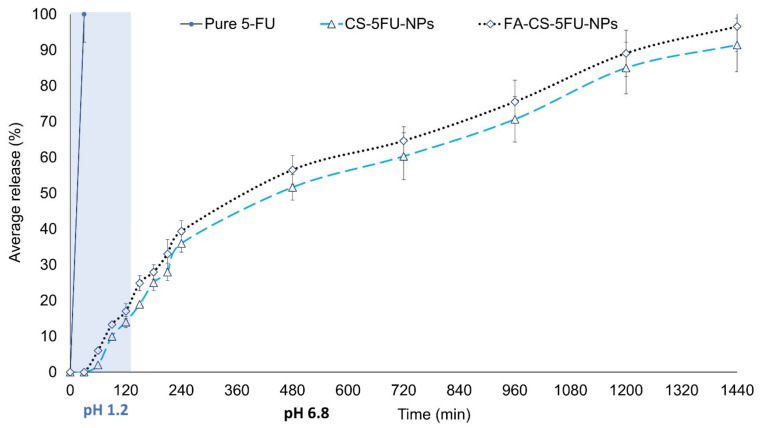
In vitro release study of pure 5-FU, CS-5FU-NPs, and FA-CS-5FU-NPs.

In vitro drug release was evaluated at a pH of 1.2 and 6.5 to measure the 5-FU release from the FA-CS-5FU-NPs and the CS-5FU-NPs using a USP dissolution apparatus 1. Such conditions were set to simulate the acidic gastric and physiological environment of the intestine. The percentage of drug released from the FA-CS-5FU-NPs and the CS-5FU-NPs was in the range of 10.08 ± 0.45% to 96.57 ± 0.09% and 6 ± 0.31% to 91.44 ± 0.21%, respectively. In artificial gastric liquid, 17.02 ± 0.12% and 14.5 ± 0.41% of 5-FU were released from the FA-CS-5FU-NPs and CS-5FU-NPs, respectively, in the first 2 h. The difference in the release pattern of these two formulations was insignificant, as shown in Figure 5 (*p* > 0.05). The initial release of 5-FU at an acidic pH was followed by a sustained release of up to 24 h. The initial release may be due to weakly bound drugs on the surface of nanoparticles [20].

Drug release at a pH of 6.5 within the first 2 h from the FA-CS-5FU-NPs and CS-5FU-NPs was 39.37 ± 3% and 36 ± 2.45%, and the accumulative release in 24 h (1440 min) was 96.57 ± 7% and 91.44 ± 7.45%, respectively. These in vitro values indicate that the FA-decorated nanoparticles can be used as a 5-FU delivery vector with a typical controlled release process. The remarkably high release rate of 5-FU from the folic-acid-conjugated nanoparticles, more interestingly at a pH of 6.5, may be due to the increased acidity of the respective release media caused by the presence of folic acid on the targeted nanoparticles. The improved hydrophilicity of the FA–CS nanoparticles due to the addition of folate was linked to the increased release rate [31]. Taken together, the acidic environments of tumor cells are likely to elicit the release of 5-FU from the developed delivery vehicles, and the sustained drug release profile from the vehicles over time can reduce dosing regimens [32].

### 3.4. Cytotoxicity Studies

**Figure 6:**
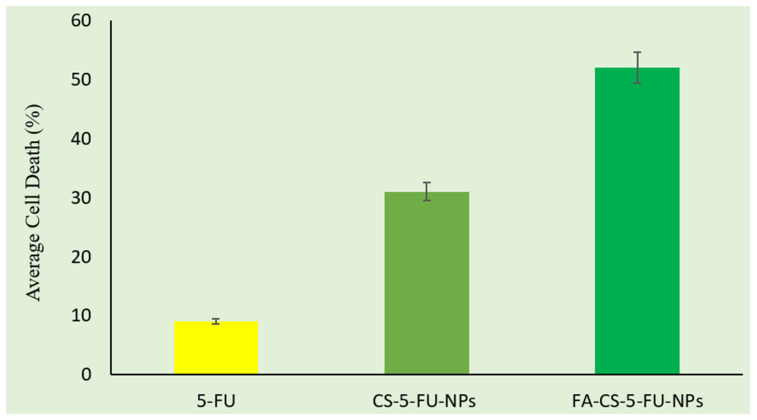
Cytotoxicity study shows the % cell death of 5-FU, CS-5-FU-NPs, and FA-CS-5-FU-NPs.

Cytotoxicity studies of free drug and NPs were performed on caco-2 cell lines. The percentage of cell death was determined and shown in Figure 5. The IC50 value of free 5-FU was found to be 4.21 µg/mL. This value was reduced to 3.43 µg/mL (CS-5-FU-NPs) and 2.67 µg/mL (FA-CS-5-FU-NPs) when 5-FU was incorporated into nanoparticles, showing significantly more cytotoxicity than the free drug. Up to 9% of cell death was induced by the free drug (5-FU solution). The percentage of cell death increased when the CS-5-FU-NPs and FA-CS-5-FU-NPs were applied. This increase might be due to the combined effect of drug and hydrophilicity of the FA–CS nanoparticles due to the addition of folate, which began to increase the release rate of 5-FU from NPs. However, a significant effect (*p* < 0.05) on the percentage of cell death was produced when the FA–CS-conjugated NPs were applied (Figure 6). This was the resultant effect of the combination of the drug and folic acid conjugation with chitosan. The FA receptors are more expressed on cancer cells; therefore, the introduction of folic acid on NPs makes them more targeted and cytotoxic in action.

## 4. Conclusions

Nanoparticles were successfully prepared using the ionic gelation method. The size and zeta potential and PDI of the CS-5FU-NPs were 208 ± 15, 26 ± 2, −20 ± 2, respectively, and those of the FA-CS-5FU-NPs were 235 ± 12, +20 ± 2 and 0.25, respectively, which are within acceptable ranges. FTIR and ^1^H-NMR studies confirmed the conjugation of folic acid with the nanoparticles. The drug contents’ % yield and the %EE of folate-decorated NPs were 53 ± 1, 80.8 and 59 ± 2%, respectively. The in vitro release of FA-CS-5FU-NPs and CS-5FU-NPs was in the range of 10.08 ± 0.45 to 96.57 ± 0.09% and 6 ± 0.31 to 91.44 ± 0.21, respectively. The percentage of cell death increased in the presence of folic acid, as compared to the free drug and chitosan nanoparticles due to the overexpression of folate receptors on the cancer cells. The results of all these parameters indicate that folate-modified chitosan 5-FU nanoparticles can be used successfully for the delivery of 5-FU with enhanced cytotoxicity and targeted delivery to the tumors.

## Figures and Tables

**Table 1:** Physicochemical characterization of folic-acid-modified 5-FU-loaded chitosan NPs. Data were presented as triplicate (*n* = 3) and mean ± SD.

Formulation Code	Size (nm)	Zeta Potential (mV)	PDI	Drug Content (%)	Percent Yield	%EE	%LE
CS-5-FU NPs	208 ± 15.00	+26 ± 2.00	0.19 ± 0.01	55 ± 1.00	90 ± 4.24	61 ± 2.00	43 ± 3.00
FA-CS-5-FU NPs	235 ± 12.00	+20 ± 2.00	0.25 ± 0.01	53 ± 1.00	80.8 ± 3.19	59 ± 2.00	39 ± 2.00

## Data Availability

Not applicable.
